# Enhancing Metastability by Dissipation and Driving in an Asymmetric Bistable Quantum System

**DOI:** 10.3390/e20040226

**Published:** 2018-03-26

**Authors:** Bernardo Spagnolo, Angelo Carollo, Davide Valenti

**Affiliations:** 1 Dipartimento di Fisica e Chimica, Group of Interdisciplinary Theoretical Physics, Università di Palermo and CNISM, Unità di Palermo, Viale delle Scienze, Edificio 18, I-90128 Palermo, Italy; 2 Istituto Nazionale di Fisica Nucleare, Sezione di Catania, I-95100 Catania, Italy; 3 Radiophysics Department, Lobachevsky State University of Nizhni Novgorod, 603000 Nizhni Novgorod, Russia; 4 Istituto di Biomedicina ed Immunologia Molecolare (IBIM) “Alberto Monroy”, CNR, Via Ugo La Malfa 153, I-90146 Palermo, Italy

**Keywords:** Caldeira-Leggett model, metastable potential, discrete variable representation, noise enhanced stability, resonant activation, tunneling, quantum Zeno dynamics, quantum systems with finite Hilbert space, functional analytical methods, open systems, quantum statistical methods

## Abstract

The stabilizing effect of quantum fluctuations on the escape process and the relaxation dynamics from a quantum metastable state are investigated. Specifically, the quantum dynamics of a multilevel bistable system coupled to a bosonic Ohmic thermal bath in strong dissipation regime is analyzed. The study is performed by a non-perturbative method based on the real-time path integral approach of the Feynman-Vernon influence functional. We consider a strongly asymmetric double well potential with and without a monochromatic external driving, and with an out-of-equilibrium initial condition. In the absence of driving we observe a nonmonotonic behavior of the escape time from the metastable region, as a function both of the system-bath coupling coefficient and the temperature. This indicates a stabilizing effect of the quantum fluctuations. In the presence of driving our findings indicate that, as the coupling coefficient γ increases, the escape time, initially controlled by the external driving, shows resonant peaks and dips, becoming frequency-independent for higher γ values. Moreover, the escape time from the metastable state displays a nonmonotonic behavior as a function of the temperature, the frequency of the driving, and the thermal-bath coupling, which indicates the presence of a quantum noise enhanced stability phenomenon. Finally, we investigate the role of different spectral densities, both in sub-Ohmic and super-Ohmic dissipation regime and for different cutoff frequencies, on the relaxation dynamics from the quantum metastable state. The results obtained indicate that, in the crossover dynamical regime characterized by damped *intrawell* oscillations and incoherent tunneling, the spectral properties of the thermal bath influence non-trivially the short time behavior and the time scales of the relaxation dynamics from the metastable state.

## 1. Introduction

The interaction between environmental random fluctuations and the nonlinearity of real systems can give rise to new unexpected phenomena. A noisy environment indeed can facilitate the permanence of a particle in a metastable region, contribute to its escape from a metastable state, or synchronize the passage between two wells in a periodically driven bistable potential. These effects are of general interest in many field of physics, chemistry and biology, where random fluctuations can accelerate or slow down physical dynamics, chemical reactions and biological processes. To describe the noise-induced synchronization (stochastic resonance) between two states [1,2,3,4,5,6,7,8,9], noise enhanced stability in metastable systems [10,11,12,13,14,15,16], stochastic resonant activation phenomenon [17,18,19,20,21,22], nonlinear relaxation with multiplicative noise [23], suppression of noise in a driven bistable system [24] and Josephson junctions [25], optimal fast single-pulse readout of qubits [26,27] and non-Gaussian anomalous diffusion [28] it is commonly used an archetypal model consisting of a particle subject to a cubic or asymmetric bistable potential and linearly coupled to a thermal bath. In quantum mechanics this model is extended in a natural way by considering a particle subject to a cubic or asymmetric bistable potential and linearly coupled to a heat bath of harmonic oscillators [29,30,31,32]. Here the time the particle takes to escape from a well characterized by a higher energy level (metastable state) towards a lower energy state depends not only on the temperature of the heat bath but also on the damping i.e., the coupling strength between the particle and the heat bath itself. Several physical and chemical processes, such as magnetization in solid state systems [33,34], proton transfer in chemical reactions [35] and superconducting devices [36,37,38], can be explained by this conceptual and mathematical description.

The decay rate has been calculated for a quantum particle moving along a cubic potential by using functional integral techniques [39,40,41]. Moreover a particle, initially placed at the bottom of a metastable well, leaves this state with a decay rate whose values decrease monotonically with the increasing damping. Conversely, an enhancement of the decay rate is found as the bath temperature increases. In addition, for a particle whose initial state is given by a Gaussian wave packet in the metastable well of a biased (asymmetric) quartic potential [42], master equation techniques allowed to predict a monotonic increase of the escape rate as a function of the temperature.

On the other side, stabilization phenomena were observed in the absence of environmental perturbations, such as the life time enhancement of a quantum metastable state driven by a suitable external periodical driving [43]. In addition, for a particle moving along a nonlinear potential driven by an external periodical signal, it has been shown that the activated escape from a metastable state can be suppressed as the temperature increases [44,45].

Environmental fluctuations are mostly considered responsible for the escape from a quantum metastable state. A fundamental question is whether the noisy environment can favour the permanence of a quantum particle in a metastable state.

This issue can be faced by taking into account a quantum particle, initially prepared in a nonequilibrium state within a strongly asymmetric bistable potential, and studying the dynamics of the spatially localized states of this system. This approach allows to observe that, as the damping increases, the relaxation process towards the stable (lower) well undergoes a modification: the population transfer, characterized by a metastable well temporarily populated, becomes a direct transfer to the stable state, due to the fact that higher damping values tend to suppress the tunnel effect in the dissipation dynamics [29,41]. The damping can therefore stabilize a quantum metastable state. In particular, the escape time from the metastable well shows a nonmonotonic behavior, with a maximum, as the damping strength increases. This indicates that for a suitable value of the damping strength the escape time takes on a maximum value, which corresponds to a *stabilization* of the quantum system, a phenomenon which can be named quantum noise enhanced stability (qNES), according to the analogous effect observed in classical physics [10,11,12,13,14,15,16,25,46,47,48,49,50,51,52,53,54,55,56,57,58,59,60,61,62,63]. This indicates that, contrary to the result predicted by Kramers [64,65], the average lifetime of a particle, initially placed in a metastable or unstable state, can be enhanced respect to the deterministic one [13,52].

In this work we shortly review some results obtained in the context of the noise induced phenomena for the system above introduced i.e., a quantum particle moving along an asymmetric bistable potential, strongly interacting within a thermal bath, with different spectral function. The transient dynamics is analyzed with and without a monochromatic external driving, and with an out-of-equilibrium initial condition.

In the absence of driving, we study the dissipative dynamics in a system consisting of a quantum particle moving along an asymmetric bistable potential, while discussing some results previously obtained within the context of the noise enhanced stability. The system dynamics is described by the well known Caldeira-Leggett model [66], in which a quantum particle, the *open system*, is linearly coupled to a *reservoir* of *N* independent quantum harmonic oscillators. The coupling between the system and each oscillator is weak (see parameter $c_{j}$ in Equation (1)). Anyway the overall damping, that is the γ parameter, due to the great number *N* of bath bosonic oscillators, can be strong, especially in macroscopic systems such as superconducting quantum interference devices [29,32]. The analysis is carried out in the thermodynamical limit (N→∞). This allows to write the spectral density function J(ω) in the form $\omega^{s}$ with a high-frequency cut-off. For s=1 the so-called Ohmic dissipation is obtained, and the quantum Langevin equation, which describes the dynamics of the particle’s position operator, is characterized by a memoryless damping kernel, which corresponds to a frequency independent damping. Under this conditions, for ℏ→0 (classical limit) the thermal bath becomes a white noise source [32].

In the presence of driving, within the framework of the dissipative quantum dynamics of the asymmetric bistable system, we study the escape dynamics from the metastable state in strong Ohmic dissipation regime. By using a monochromatic signal with a suitable amplitude, we analyze the escape time as a function of the driving frequency. The study shows the presence of resonant peaks and dips for lower values of the coupling coefficient γ. Conversely, as γ increases, the escape time displays a maximum and then a rapid decrease. In particular, a value of the coupling parameter $\gamma_{c}$ exists such that for $\gamma >\gamma_{c}$ the escape time becomes frequency-independent, resembling the static case and suggesting to interpret $\gamma_{c}$ as a critical value, in correspondence of which a qualitatively different escape dynamics can be observed.

Finally, the role of different spectral densities, both in sub-Ohmic and super-Ohmic dissipation regime and for different cutoff frequencies, on the relaxation dynamics from the quantum metastable state is investigated. The results obtained indicate that, in the crossover dynamical regime characterized by damped *intrawell* oscillations and incoherent tunneling, the spectral properties of the thermal bath influence non-trivially the short time behavior and the time scales of the relaxation dynamics from a metastable state.

## 2. Model

**Figure 1:**
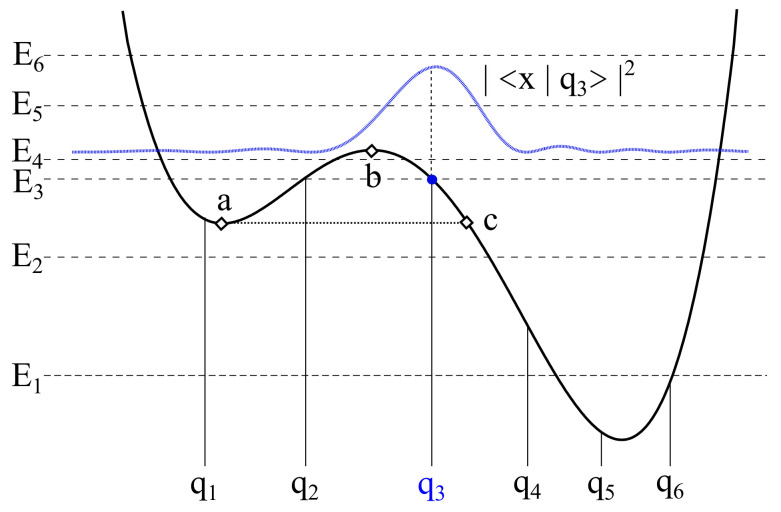
(Color online) Potential V(q) (Equation (2)) for $\Delta U=1.4\;\hbar \omega_{0}$ and $\epsilon =0.27\sqrt{M\hbar \omega_{0}^{3}}$. Horizontal lines: the first 6 energy levels. Vertical lines: the position eigenvalues in the DVR. The blue solid curve is the initial probability density ${\left|\Psi \left(x,0\right)\right|}^{2}$. For the tunneling splitting we have $\Delta E_{4,3}=E_{4}-E_{3}=0.2\;\hbar \omega_{0}$ while $E_{2}-E_{1}=0.985\;\hbar \omega_{0}$. The initial condition $q_{3}$ is the blue point.

For a quantum *particle*, which moves in a double well potential (see Figure 1), the total Hamiltonian is

(1) $$\begin{array}{c} \hat{H}=\frac{{\hat{p}}^{2}}{2M}+V\left(\hat{q}\right)+\sum_{j=1}^{N}\frac{1}{2}\left[\frac{{\hat{p}}_{j}^{2}}{m_{j}}+m_{j}\omega_{j}^{2}{\left({\hat{x}}_{j}-\frac{c_{j}}{m_{j}\omega_{j}^{2}}\hat{q}\right)}^{2}\right]={\hat{H}}_{0}+{\hat{H}}_{B+SB}\;, \end{array}$$

where ${\hat{H}}_{0}={\hat{p}}^{2}/2M+V\left(\hat{q}\right)$ is the system’s bare Hamiltonian, ${\hat{H}}_{B+SB}$ is the bath Hamiltonian including the interaction with the system, and

(2) $$V\left(\hat{q}\right)=\frac{M^{2}\omega_{0}^{4}}{64\Delta U}{\hat{q}}^{4}-\frac{M\omega_{0}^{2}}{4}{\hat{q}}^{2}-\hat{q}\epsilon \;,$$

Is the asymmetric bistable potential. In Equation (1), the constant $c_{j}$ measures the coupling of the particle with the j-th oscillator. The renormalization term quadratic in $\hat{q}$ is introduced to give a purely dissipative bath, that is a spatially homogeneous dissipation [32]. In Equation (2), *M* is the effective mass, $\omega_{0}$ the natural oscillation frequency around the minima (which is of the same order of magnitude of the frequency spacing between the ground state’s energy level and the first excited energy level), ΔU the barrier height, and ϵ the asymmetry parameter. Here ϵ is large enough that the potential of Equation (2) can be treated as a cubic potential, which allows to treat in an effective way the metastable state dynamics and escape problems. The interaction with the environment occurs through the coupling with a thermal bath of *N* independent harmonic oscillators with position coordinates ${\hat{x}}_{j}$. Each oscillator is coupled with the particle through a linear interaction given by $\sum_{j=1}^{N}c_{j}{\hat{x}}_{j}\hat{q}$. In the continuum limit N→∞, we consider for the dissipative environment the Ohmic spectral density function *J* with a high-frequency cutoff [32]

(3) $$J\left(\omega \right)=M\gamma \omega e^{-\omega /\omega_{c}}.$$

The cut-off frequency $\omega_{c}$ is chosen in such a way to be much larger than all the other frequencies involved in the dynamics, that is $\omega_{0}$ and the frequencies corresponding to jumps between different energy levels of the static potential (see Figure 1). The damping coefficient γ accounts for the overall particle-bath coupling strength according to the classical damping in the quantum Langevin equation, which can be written for this problem. Moreover, the damping coefficient is frequency independent in the case of Ohmic bath [32]. If the damping parameter γ is sufficiently below the frequency spacing between ground state and the first excited energy level ($≃\omega_{0}$) a description of the state of the system in terms of unperturbed energy levels is viable.

Due to the initial state of the particle and the value fixed for the bath temperature (which fulfills the condition $k_{B}T/\hbar \ll \omega_{0}$), the dynamics is de facto limited to the first 6 levels of the potential shown in Figure 1. This reduced Hilbert space allows to pass, by suitably transforming, to the discrete variable representation (DVR) [67]. The particle’s time evolution is obtained, by tracing off the freedom degrees of the bath, in terms of reduced dynamics in the *localized* basis of the position eigenstates $\{|q_{1}\rangle ,\ldots ,|q_{6}\rangle \}$, with $\hat{q}|q_{i}\rangle =q_{i}|q_{i}\rangle$. From here onwards, the parameters γ and *T* are given in units of $\omega_{0}$ and $\hbar \omega_{0}/k_{B}$, respectively.

## 3. The Influence Functional and the Discrete Variable Representation

We assume that the particle-bath total density matrix η(t) is initially in the factorized form

(4) $$\eta \left(t_{0}\right)=\rho \left(t_{0}\right)⊗\frac{e^{\beta H_{B}}}{Z_{B}}=\rho \left(t_{0}\right)⊗\frac{e^{\beta H_{B}}}{Tr_{B}e^{\beta H_{B}}}\;,$$

where $H_{B}=\sum_{j=1}^{N}\left(1/2\right)\left[{\hat{p}}_{j}^{2}/m_{j}+m_{j}\omega_{j}^{2}{\hat{x}}_{j}^{2}\right]$ is the free bath Hamiltonian, $\rho (t_{0})$ is the initial particle density matrix of the system and the bath is in thermal equilibrium at temperature $T=1/(\beta k_{B})$.

The time evolution of the particle’s reduced density matrix, obtained by performing the partial trace over the bath’s degrees of freedom on η(t), evolves according to [32]

(5) $$\rho \left(q_{f},q_{f}^{\prime },t\right)=\int dq_{0}\int dq_{0}^{\prime }G\left(q_{f},q_{f}^{\prime },t;q_{0},q_{0}^{\prime },t_{0}\right)\rho \left(q_{0},q_{0}^{\prime },t_{0}\right),$$

The propagator $G(q_{f},q_{f}^{\prime },t;q_{0},q_{0}^{\prime },t_{0})$ can be put in the form of a double path integral in which the contribution of the bath and that of the external potential to the evolution are factorized [68]

(6) $$G\left(q_{f},q_{f}^{\prime },t;q_{0},q_{0}^{\prime },t_{0}\right)=\int_{q\left(t_{0}\right)=q_{0}}^{q\left(t\right)=q_{f}}Dq\left(t\right)\int_{q^{\prime }\left(t_{0}\right)=q_{0}^{\prime }}^{q^{\prime }\left(t\right)=q_{f}^{\prime }}D^{*}q^{\prime }\left(t\right)A\left[q\right]A^{*}\left[q^{\prime }\right].F_{FV}\left[q\left(t\right),q^{\prime }\left(t\right)\right],$$

where the integration symbol in Equation (6) is defined as [32,69]

$$\int Dq=\lim_{N\to \infty }\sqrt{\frac{M}{i2\pi \hbar \Delta t}}\prod_{n=1}^{N-1}dq_{n},$$

with $\Delta t=(t-t_{0})/N$ the size of each time interval in which the total time interval of the propagator $t-t_{0}$ is divided in N≫1 intervals. In Equation (6) $A\left[q\left(t\right)\right]=e^{\frac{i}{\hbar }S_{S}^{0}\left[q\left(t\right)\right]}$ is the amplitude associated with the path q(t) of the bare system, where $S_{S}^{0}$ is the action relative to the particle subject solely to the external force. The effect of the bath is taken into account by the Feynman-Vernon (FV) influence functional $F_{FV}\left[q\left(t\right),q^{\prime }\left(t\right)\right]=e^{-\frac{1}{\hbar }\Phi_{FV}\left[q\left(t\right),q^{\prime }\left(t\right)\right]}$ [68]. The exponent $\Phi_{FV}\left[q\left(t\right),q^{\prime }\left(t\right)\right]$, named influence phase functional, contains information on the bath properties through the function Q(t), related to the bath correlation function L(t) by $L\left(t\right)=\hbar^{2}d^{2}Q\left(t\right)/dt^{2}$. The expressions of the bath correlation function L(t) and function Q(t) are respectively given by [32,69]

(7) $$L\left(t\right)=L^{\prime }\left(t\right)+iL"\left(t\right)=\frac{\hbar }{\pi }\int_{0}^{\infty }d\omega J\left(\omega \right)\left(\coth\frac{\hbar \omega \beta }{2}\cos\omega t-i\sin\omega t\right),$$

(8) $$Q\left(t\right)=S\left(t\right)+iR\left(t\right)=\frac{1}{\pi \hbar }\int_{0}^{\infty }d\omega \frac{J(\omega )}{\omega^{2}}\left\{\coth\frac{\hbar \omega \beta }{2}\left(1-\cos\omega t\right)+i\sin\omega t\right\},$$

where the spectral density J(ω) is given by Equation (3). In the scaling limit set by $k_{B}T/\hbar \omega_{c}\ll 1$, we have [69]

(9) $$Q\left(t\right)=\frac{M\gamma }{\pi \hbar }\ln\left(\sqrt{1+\omega_{c}^{2}t^{2}}\frac{\sinh(\kappa t)}{\kappa t}\right)+i\frac{M\gamma }{\pi \hbar }\arctan\left(\omega_{c}t\right),$$

where $\kappa =\pi k_{B}T/\hbar$. The long time or moderately high temperature limit $(k_{B}T/\hbar \omega_{c})t\gg 1$ of Equation (9) has a linear dependence on time [32,69]

(10) $$Q\left(t\right)≃\frac{M\gamma }{\pi \hbar }\left[\frac{\pi }{\hbar \beta }t-\ln\left(\frac{2\pi }{\beta \hbar \omega_{c}}\right)\right]+i\frac{M\gamma }{2\hbar }.$$

Once specified the parameters we make a restriction on the dimensionality of the relevant particle’s Hilbert space by confining ourselves to the first 6 energy levels $\left\{E_{1},\ldots ,E_{6}\right\}$. The underlying assumption is that the system is not going to be excited to energies higher than $E_{6}$. Performing on this restricted basis the unitary transformation T, which diagonalizes the position operator $\hat{q}$ of matrix elements $<E_{i}\left|\hat{q}\right|E_{j}>$, we pass to the discrete variable representation (DVR) [67,70,71]. The 6 position eigenfunctions with eigenvalues $\left\{q_{0},\ldots ,q_{L-1}\right\}$, obtained by performing a suitable transformation on the basis set $\left\{|E_{i}\rangle \right\}$, provide the Discrete Variable Representation (DVR).

In this representation a path consists of a sequence of transitions in the spatial grid defined by the set $\left\{q_{1},\ldots ,q_{6}\right\}$ so that the double path integral (6) turns into a sum over all the possible discrete paths $\left\{\mu_{j},\nu_{j}\right\}$ with transitions at times $\left\{t_{1},t_{2}\ldots ,t_{m}\right\}$, integrated over the times $\left\{t_{j}\right\}$ and summed over all the possible numbers *m* of transitions. The time evolutions of the diagonal elements $\rho \left(q_{\mu },q_{\mu },t\right)\equiv \rho_{\mu \mu }\left(t\right)$ are thus given by

(11) $$\begin{array}{cc} \rho_{\mu \mu }\left(t\right)= & \sum_{\mu_{0},\nu_{0}=1}^{6}\rho_{\mu_{0},\nu_{0}}\left(t_{0}\right)\sum_{m=1}^{\infty }\{\int_{t_{0}}^{t}dt_{m}\int_{t_{0}}^{t_{m}}dt_{m-1}\ldots \int_{t_{0}}^{t_{2}}dt_{1}\\ & \sum_{\left\{paths\right\}}e^{\frac{i}{\hbar }S\left[q_{\mu_{j}}\right]-\frac{i}{\hbar }S\left[q_{\nu_{j}}\right]}F_{FV}\left[\xi \left(t_{j}\right),\chi \left(t_{j}\right)\right]\}\;\left(j=1,\ldots ,m\right), \end{array}$$

where the relative coordinate $\xi \left(t_{j}\right)=q_{\mu_{j}}-q_{\nu_{j}}=q\left(t_{j}\right)-q^{\prime }\left(t_{j}\right)$ and the center of mass coordinate $\chi \left(t_{j}\right)=q_{\mu_{j}}+q_{\nu_{j}}=q\left(t_{j}\right)+q^{\prime }\left(t_{j}\right)$ are used.

In terms of ξ and χ the influence phase functional for a set of paths, each consisting of *m* transitions $\left(\mu ,\nu \right)\to \left(\mu^{\prime },\nu^{\prime }\right)$ at times $\left\{t_{1},t_{2}\ldots ,t_{m}\right\}$, has the following explicit form

(12) $$\Phi_{FV}\left[\xi \left(t_{j}\right),\chi \left(t_{j}\right)\right]=-\sum_{i=1}^{m}\sum_{l=0}^{i-1}\xi_{i}S\left(t_{i}-t_{l}\right)\xi_{l}-i\sum_{i=1}^{m}\sum_{l=0}^{i-1}\xi_{i}R\left(t_{i}-t_{l}\right)\chi_{l},$$

where $\xi_{i}=\xi \left(t_{i}\right)-\xi \left(t_{i-1}\right)$ and $\chi_{i}=\chi \left(t_{i}\right)-\chi \left(t_{i-1}\right)$ ($\xi_{0}\equiv \xi \left(t_{0}\right)$ and $\chi_{0}\equiv \chi \left(t_{0}\right)$) are termed *charges*. $S(t_{i}-t_{l})$ and $R(t_{i}-t_{l})$ are defined in (8).

As a further approximation, in addition to the DVR, we restrict the sum over paths in the propagator *G* to the leading contributions. These are given by the class of paths consisting in sojourns in diagonal states interrupted by single off-diagonal excursions called *blips*. In the dissipation regimes from intermediate to high temperature, on the scale fixed by $\hbar \omega_{0}$, considered here, the time nonlocal interactions among different blips in Equation (12) (*inter-blip* interactions) can be neglected. This corresponds to a multilevel version [71,72,73,74] of the *non-interacting blip approximation* (NIBA) [30,32]. However, the relevant part of the interactions, the intra-blip interactions are retained to all orders in the coupling strength. Since the total sum of the charges in a path connecting two diagonal elements is zero, all the interactions between different paths connecting two diagonal elements ($\rho_{\mu \mu }\left(t_{1}\right)↔\rho_{\nu \nu }\left(t_{2}\right)$) are neglected. This is the content of the generalized *non-interacting cluster approximation* (gNICA) [72], where a *cluster* is defined as the time interval during which the path visits non-diagonal elements. The gNICA is the generalization to a multilevel system of the NIBA applicable for a spin-boson system [72].

## 4. Master Equation

Within the gNICA the time integrations in Equation (11) take the form of convolutions. As a consequence, the path integral expression for a population (diagonal elements of the reduced density matrix) in Laplace space takes the form of a series in the number of transitions. The series is resummed and by performing the inverse Laplace transform we get in the time domain a generalized master equation (GME). If ρ(0) is diagonal in the position representation, the GME reads

(13) $${\dot{\rho }}_{\mu \mu }\left(t\right)=\sum_{\nu =1}^{6}\int_{0}^{t}dt^{\prime }K_{\mu \nu }^{\left(2\right)}\left(t-t^{\prime }\right)\rho_{\nu \nu }\left(t^{\prime }\right)+I_{\mu }\left(t-t_{0}\right).$$

In Equation (13) the overdot denotes the derivative with respect to time *t*. Moreover, the second order transition probability (involving only one intermediate step) for the process $\rho_{\nu \nu }\left(t^{\prime }\right)\to \rho_{\mu \mu }\left(t\right)$ through the single non-diagonal state $\rho_{\mu \nu }$ ($\rho_{\nu \mu }$), in the general case of the presence of an external periodical driving, is given by

(14) $$\begin{array}{c} K_{\mu \nu }^{\left(2\right)}\left(t-t^{\prime }\right)=\frac{{\left[\langle q_{\mu }|{\hat{H}}_{0}|q_{\nu }\rangle \right]}^{2}}{2}\exp\left[-{\left(q_{\mu }-q_{\nu }\right)}^{2}S\left(t-t^{\prime }\right)\right]\;\;\left(\mu \neq \nu \right)\\ \;\times \cos\left[\int_{t^{\prime }}^{t}dt^{\prime \prime }\left(\langle q_{\nu }|{\hat{H}}_{S}\left(t^{\prime \prime }\right)|q_{\nu }\rangle -\langle q_{\mu }|{\hat{H}}_{S}\left(t^{\prime \prime }\right)|q_{\mu }\rangle \right)-{\left(q_{\mu }-q_{\nu }\right)}^{2}R\left(t-t^{\prime }\right)\right]\;\;\; \end{array}$$

where ${\hat{H}}_{0}=\frac{{\hat{p}}^{2}}{2M}+\hat{V}\left(\hat{q}\right)$ and ${\hat{H}}_{S}\left(t\right)={\hat{H}}_{0}-\hat{q}A\sin\Omega t$. In the absence of driving we have A=0 and the matrix elements inside the integral are time independent. The kernel for the process $\rho_{\nu \nu }\left(t^{\prime }\right)\to \rho_{\nu \nu }\left(t\right)$ is given by the conservation of probability

(15) $$K_{\nu \nu }^{\left(2\right)}\left(t-t^{\prime }\right)=-\sum_{\lambda \neq \nu }^{6}K_{\lambda \nu }^{\left(2\right)}\left(t-t^{\prime }\right).$$

The inhomogeneity term $I_{\mu }\left(t-t_{0}\right)$ in (13) arises because of the contributions of the non-diagonal initial states, which contain the coherences at initial time $t_{0}$. This term of inhomogeneity vanishes when the non-diagonal elements of the particle’s density matrix at $t=t_{0}$ are zero. Here we choose as initial condition (see Equation (11)) $\rho \left(t_{0}\right)=|q_{i}\rangle \langle q_{i}|$, so that initially non-diagonal elements of the particle’s density matrix vanish and therefore the term $I_{\mu }\left(t-t_{0}\right)$ is equal to zero. Moreover, this term $I_{\mu }\left(t-t_{0}\right)$ is exponentially damped on a time scale determined by the damping constant γ and the temperature *T*. By investigating the long-time dynamics, in the case of non-diagonal initial states different from zero, $I_{\mu }\left(t-t_{0}\right)$ can be neglected and only the populations remain involved in the GME [72]. Therefore, we have in both cases

(16) $${\dot{\rho }}_{\mu \mu }\left(t\right)=\sum_{\nu =1}^{6}\int_{0}^{t}dt^{\prime }K_{\mu \nu }\left(t-t^{\prime }\right)\rho_{\nu \nu }\left(t^{\prime }\right).$$

The kernel elements $K_{\mu \nu }$ are taken to the second order in the transition amplitudes per unit time $\Delta_{ij}=\langle q_{i}|{\hat{H}}_{0}|q_{j}\rangle /\hbar$ (see Equation (14)) and at all orders in the system-bath coupling. They go to zero exponentially due to the presence of a cut-off. At strong damping, as in our case, in the short time interval in which $K_{\mu \nu }$ are substantially different from zero, $\rho_{\nu \nu }\left(t\right)$ are practically constant. Under the assumption (Markovian approximation) that $\rho_{\nu \nu }\left(t^{\prime }\right)$ in (16) are practically constant on the time scales at which the kernels $K_{\mu \nu }$ are significantly different from zero, we can put them outside the integral and bring the upper limit to *∞*. Setting $t_{0}=0$, the time-independent rates are thus given by

(17) $$\Gamma_{\mu \nu }=\int_{0}^{\infty }d\tau K_{\mu \nu }\left(\tau \right),$$

where the kernels $K_{\mu \nu }\left(\tau \right)$ are given by Equation (14) with A=0 (absence of driving). Under these assumptions we get

(18) $${\dot{\rho }}_{\mu \mu }\left(t\right)=\sum_{\nu =1}^{6}\Gamma_{\mu \nu }\rho_{\nu \nu }\left(t\right),$$

which is the Markov approximated version of Equation (16).

Furthermore, if the driving frequency is high enough, (ℏΩ much larger than the energy differences between doublets associated to the tunneling, $\Omega ≳\omega_{0}$) we can average the resulting (time dependent) transition coefficients over a driving period so that the final master equation in the presence of driving takes the form [72]

(19) $${\dot{\rho }}_{\mu \mu }\left(t\right)=\sum_{\nu =1}^{6}\Gamma_{\mu \nu }^{av}\rho_{\nu \nu }\left(t\right)$$

where, setting $t_{0}=0$, the time independent averaged rate coefficients are

(20) $$\begin{array}{cc} \Gamma_{\mu \nu }^{av} & =\int_{0}^{\frac{2\pi }{\Omega }}dt\int_{0}^{\infty }d\tau K_{\mu \nu }^{\left(2\right)}\left(t,\tau \right)\\ & =\frac{{\left[\langle q_{\mu }|{\hat{H}}_{0}|q_{\nu }\rangle \right]}^{2}}{2}\int_{0}^{\infty }d\tau \exp\left[-{\left(q_{\mu }-q_{\nu }\right)}^{2}S\left(\tau \right)\right]J_{0}\left[\frac{2s}{\Omega }\left(q_{\mu }-q_{\nu }\right)\sin\left(\frac{\Omega }{2}\tau \right)\right]\\ & \;\times \cos\left[\langle q_{\nu }|{\hat{H}}_{0}|q_{\nu }\rangle -\langle q_{\mu }|{\hat{H}}_{0}|q_{\mu }\rangle )\tau -{\left(q_{\mu }-q_{\nu }\right)}^{2}R\left(\tau \right)\right]\;\;\mu \neq \nu \\ \Gamma_{\nu \nu }^{av} & =-\sum_{\lambda \neq \nu }^{6}\Gamma_{\lambda \nu }^{av}. \end{array}$$

### Solution

The solution of (19) is

(21) $$\rho_{\mu \mu }\left(t\right)=\sum_{\lambda ,\nu =1}^{6}S_{\mu \lambda }{\left(S^{-1}\right)}_{\lambda \nu }e^{\Lambda_{\lambda }t}\rho_{\nu \nu }\left(0\right)$$

where *S* is the transformation matrix giving the eigenvalues $\Lambda_{\lambda }$ of the rate matrix $\Gamma^{av}$ by means of

(22) $$\Lambda_{\lambda }\delta_{\lambda \nu }=\sum_{\alpha ,\beta =1}^{6}{\left(S^{-1}\right)}_{\lambda \alpha }\Gamma_{\alpha \beta }^{av}S_{\beta \nu }$$

The condition $\Gamma_{\nu \nu }^{av}=-\sum_{\lambda \neq \nu }^{6}\Gamma_{\lambda \nu }^{av}$ implies that $\Lambda_{0}=0$, so that the corresponding coefficient $\sum_{\lambda =1,\nu =0}^{6}S_{\mu \lambda }{\left(S^{-1}\right)}_{\lambda \nu }\rho_{\nu \nu }\left(0\right)$ is the asymptotic value $\rho_{\mu \mu }^{\infty }$ of the population $\rho_{\mu \mu }$. The smallest, in absolute value, of the remaining $\Lambda_{\lambda }$ determines the largest time-scale of the dynamics of $\rho_{\mu \mu }\left(t\right)$, that is the *quantum relaxation time* $\tau_{relax}$ defined as $\Lambda_{min}^{-1}$.

Indeed, this theoretical technique is non-perturbative in the system-bath coupling, and is thus suited to deal with strong coupling regime. However, the FV influence functional makes the path integral intractable as it introduces time nonlocal *interactions* between the paths q(t) and q′(t) through the bath correlation function Q(t). The nonlocal part of the interactions cancels out in the limit in which the bath correlation function Q(t) is linear in *t* (see Equation (10)), i.e., in the long time limit $t\gg \hbar /k_{B}T$. For sufficiently high temperature, Q(t) can be taken in the linearized form at all times, and this amounts to perform the so called *generalized non-interacting cluster approximation* gNICA [72], the multi-level version of the NIBA used for the spin-boson model [29,32]. If we compare the transition probabilities per unit time among the $|q_{i}\rangle$’s with $k_{B}T/\hbar$, we obtain the inequality $T≳0.1\;\hbar \omega_{0}/k_{B}$ to have a rough estimate for the validity of the gNICA for our system.

## 5. Transient Dynamics in the Absence of Driving

Now we consider the transient dynamics of the quantum particle, as given by Equation (21), with the nonequilibrium initial condition

(23) $$\rho \left(0\right)=|q_{3}\rangle \langle q_{3}|,$$

That is with the particle’s probability density initially peaked on the right of the potential barrier, in the interval ($q_{b},q_{c}$), where $q_{c}$ is the *exit point* (see Figure 1 and Reference [32]). This may be experimentally attained by preparing the particle in the ground state of an appropriate harmonic well centered at the desired position, and then releasing the harmonic potential by rapidly modifying its profile [75].

We note that the transient dynamics here considered is qualitatively different from that usually investigated, for example, in References [39,40,41,42,43,44,45]. There, the calculated decay rate gives information on the time the particle takes to leave the metastable well. Specifically in Reference [41] and references therein, the particle is initially in the ground state of a metastable cubic potential. The thermodynamical method used there [41] is not suited to treat out of equilibrium dynamics, as we do in this work. For this purpose we introduce an approach based on the escape time which is suitable to describe out-of-equilibrium dynamics in asymmetric bistable quantum systems, closely resembling the escape problems typical of the classical statistical physics [10,13,15].

Processes starting from nonequilibrium initial conditions are commonly encountered in nature, at the classical and quantum scale (see, for example, References [76,77] and references therein). A typical example of nonequilibrium dynamics is that emerging from a sudden quenching [77,78,79,80,81,82,83].

We introduce the escape time τ from the *metastable region*, defined as the region to the left of the *exit point* (point *c* in Figure 1), according to Reference [42]. There, the decay rate from the metastable region is calculated by using the probability of penetration of the Gaussian wave packet from left to right through the potential barrier of Figure 1. Here, we use a discretized version of this theoretical technique. Therefore, we calculate the population of the lower (right side) well, that is the cumulative population of the three DVR states from $|q_{4}\rangle$ to $|q_{6}\rangle$

(24) $$P_{\mathrm{right}}\left(t\right)=\sum_{\mu =4}^{6}\rho_{\mu \mu }\left(t\right).$$

During the transient dynamics the populations of the metastable states ($|q_{1}\rangle$ and $|q_{2}\rangle$) reach a maximum Afterwards, by tunneling through the potential barrier, the population of the metastable well decays, finally settling down to a stationary value dependent on the temperature. We note that actually we calculate the escape time from the *metastable region* (see Figure 1 and page 190 of Reference [32]), therefore comprising the metastable well.

We consider a large asymmetry of the potential, low temperatures with respect to the barrier height, and damping regimes ranging from moderate to strong ($\gamma ≳\omega_{0}$). Given the above conditions, the relaxation occurs in the incoherent regime, with no oscillations in the populations [72]. As a consequence, we may consider the particle irreversibly out from the metastable region once $P_{\mathrm{right}}\left(t\right)$ has reached a certain threshold value that we set at $P_{\mathrm{right}}\left(\tau \right)=0.95$.

By choosing this threshold we mean that we consider the particle escaped from the metastable region when the probability to detect it in the lower (right) well is equal or greater than 95%. Due to the incoherent relaxation described by Equation (21), once the threshold is crossed no oscillatory behavior of the populations occurs, that is no re-crossing of the threshold in the opposite direction is possible. Therefore, if the particle crosses the threshold at time τ, this means that after the time τ the particle can be considered “escaped” and the overall population of the metastable region at later times will not be larger than 0.05.

### 5.1. Results

**Figure 2:**
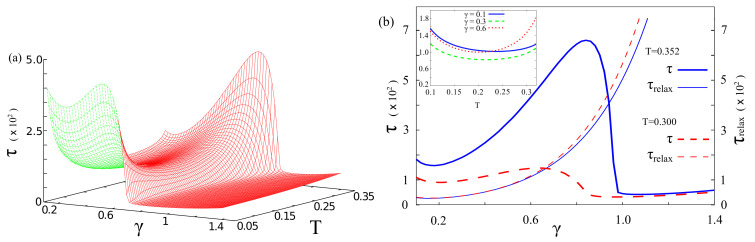
(Color online) Escape time τ, in units of $\omega_{0}^{-1}$, for the initial condition $\rho \left(0\right)=|q_{3}\rangle \langle q_{3}|$ (see Equation (23)). (**a**) τ as a function of both damping γ and temperature *T*, with threshold 0.95; (**b**) τ and $\tau_{\mathrm{relax}}$ as functions of γ for different temperatures, namely T=0.3,0.352. Inset: Escape time vs. temperature at fixed values of damping, namely γ=0.1,0.3,0.6. The parameters γ and *T* are given in units of $\omega_{0}$ and $\hbar \omega_{0}/k_{B}$, respectively. The potential parameters are the same as in Figure 1, namely $\Delta U=1.4\;\hbar \omega_{0}$ and $\epsilon =0.27\sqrt{M\hbar \omega_{0}^{3}}$.

In Figure 2 it is shown the nonmonotonic behavior of the escape time τ as a function of the coupling parameter, or damping, γ and the temperature *T*.

Specifically the behavior of τ versus γ shows a maximum, whose height and position depend on the temperature. A comparison between τ and $\tau_{\mathrm{relax}}$ versus γ indicates that the two quantities exhibit roughly the same behavior until the peak in τ is reached (see Figure 2b). At higher γ, while $\tau_{\mathrm{relax}}$ continues to increase monotonically, τ has a sudden fall off at a critical value $\gamma_{c}$, dependent on the temperature (for example $\gamma_{c}≃0.98$ at T=0.352) (see the end of Section 2 for the units of these quantities).

This critical value corresponds to a dynamical regime in which the population transfer from the initial state to the states of the metastable well is inhibited, with a direct transfer occurring to the states of the lower right well. In this regime the probability of finding the particle in the metastable region is negligibly small throughout the entire dynamics. Indeed, while $\tau_{\mathrm{relax}}$ is the time needed for the system to reach the equilibrium in the double well potential, the escape time is a relevant quantity for the transient dynamics, involving the crossing of the potential barrier and the emptying of the metastable well. Therefore, our analysis applies to the general problem of the escape from a metastable well, starting from a nonequilibrium condition. We note that applying the same technique used in classical systems to calculate the nonlinear relaxation time (NLRT) [11,25,84], at fixed temperature, we get the monotonic behavior shown in Figure 2b, which resembles that of $\tau_{\mathrm{relax}}$ versus γ (not shown here). In other words the NLRT corresponds to $\tau_{\mathrm{relax}}$.

The nonmonotonic behavior of τ vs. γ can be interpreted as the quantum counterpart of the NES phenomenon observed in classical systems, and may be called quantum noise enhanced stability (qNES) [85].

Another interesting feature is the presence of a slow monotonic increase of τ for $\gamma >\gamma_{c}$, which leads to the quantum Zeno effect [86,87,88,89,90,91,92]. It is worthwhile to note that this effect is not restricted to the measurement process but it is possible to freeze a system in a state, or an interesting subspace of the state space of the system. This type of control can be achieved through three kinds of interaction: (i) frequent measurements; (ii) quantum dynamical decoupling; (iii) strong continuous coupling. The coupling with a noisy environment (thermal bath) can be seen as a particular case of the third case [90].

The behavior of τ vs. the temperature is characterized by a minimum as $k_{B}T$ approaches the tunneling splitting $\Delta E_{4,3}=E_{4}-E_{3}=0.2\;\hbar \omega_{0}$ (see Figure 1). This is the signature of the *thermally activated tunneling*, an experimentally well established phenomenon [93]. This is better shown in the inset of Figure 2b.

**Figure 3:**
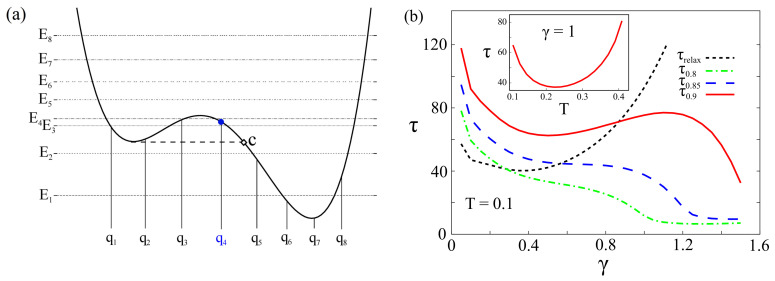
(Color online) (**a**) Potential V(q) ($\Delta U=1.4\;\hbar \omega_{0}$ and $\epsilon =0.27\sqrt{M\hbar \omega_{0}^{3}}$, the same as in Figure 1) with the first M=8 energies levels $E_{i}$ (horizontal lines) and corresponding position eigenvalues $q_{i}$ in the DVR (vertical lines); (**b**) Escape time τ at various thresholds (0.8,0.85,0.9) and relaxation time $\tau_{\mathrm{relax}}$, both in units of $\omega_{0}^{-1}$, vs. the damping strength γ at T=0.1. Inset: τ vs. *T* at γ=1. The particle is initially in the state $|q_{4}\rangle$, i.e., *localized* around $q_{4}$ (blue point in (**a**)). The parameters γ and *T* are given in units of $\omega_{0}$ and $\hbar \omega_{0}/k_{B}$, respectively.

**Figure 4:**
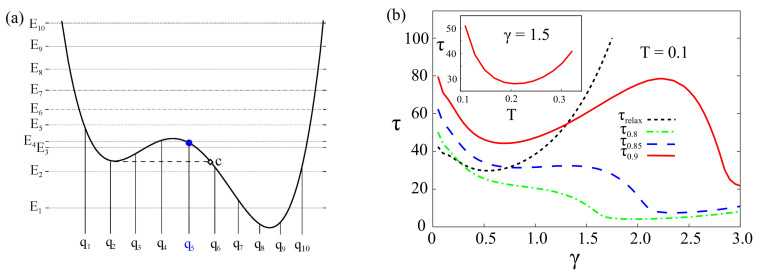
(Color online) (**a**) Potential V(q) ($\Delta U=1.4\;\hbar \omega_{0}$ and $\epsilon =0.27\sqrt{M\hbar \omega_{0}^{3}}$, the same as in Figure 1) with the first M=10 energies levels $E_{i}$ (horizontal lines) and corresponding position eigenvalues $q_{i}$ in the DVR (vertical lines); (**b**) Escape time τ at various thresholds (0.8,0.85,0.9) and relaxation time $\tau_{\mathrm{relax}}$, both in units of $\omega_{0}^{-1}$, vs. the damping strength γ at T=0.1. Inset: τ vs. *T* at γ=1.5. The particle is initially *localized* around $q_{5}$ (blue point in (**a**)). The parameters γ and *T* are given in units of $\omega_{0}$ and $\hbar \omega_{0}/k_{B}$, respectively.

We wish to point out that our results are robust against the variation of the potential asymmetry, threshold value, initial conditions, chosen within the interval ($q_{b},q_{c}$) (see Figure 3 and Figure 4), and the dimension of the reduced Hilbert space of the system [85]. The path integral approach within the discrete variable representation is not spatially continuous: the spatial continuity is recovered in the limit of an infinite number of energy levels. Nevertheless, by increasing gradually the number *M* of energy states taken into account in our approximation of *M*-state system, the DVR states change their “localization” and become more dense, especially in the regions where the potential energy is lower (inside the two wells). This means that enlarging the Hilbert space considered, new DVR states with different eigenvalues in the interval ($q_{b},q_{c}$) can be used as initial conditions.

In what follows, we show that the escape time τ as a function of the threshold values, the number *M* of energy states considered, and the initial localization of the particle, follows a behaviour qualitatively similar to that exhibited as a function of γ and *T* (see Figure 2). The results obtained by considering the same potential profile as in Figure 1, with potential parameters $\Delta U=1.4\;\hbar \omega_{0}$ and $\epsilon =0.27\sqrt{M\hbar \omega_{0}^{3}}$, are shown in Figure 3 and Figure 4. We note that, by fixing the potential profile and changing the number of energy levels, it has the effect of modifying the spatial configurations of the DVR states. This, in turn, allows us to consider new initial conditions with localized wave packets within the interval of interest, leaving the potential unchanged. Therefore, we change the number of energy levels and, as a consequence, the spatial configurations of the DVR states, the initial conditions and the threshold values. Specifically we consider M=8,10 and, in each of these cases, the escape time is defined with the three thresholds 0.8, 0.85 and 0.9. A nonmonotonic behavior of the escape time with respect to the damping parameter, similar to that described above, is observed in all considered cases (see Figure 3 and Figure 4). The minima in the *T*-dependence are also present for M=8, 10 at $T∼0.21\;\hbar \omega_{0}/k_{B}$, corresponding to the tunneling splitting $E_{4}-E_{3}=0.2\;\hbar \omega_{0}$. Finally, we note that the qNES effect is due to both tunneling effect and transitions from low level states inside the metastable state to higher level states over the potential barrier. This means that it should be observed also for larger number of levels inside the metastable potential well.

## 6. Driven Quantum Dissipative Dynamics

As a model of driven dissipative quantum dynamics confined between two metastable wells, we modify the bistable system used in Section 2, by considering an external periodical driving. Therefore, the open system *S* is now given by a quantum particle of effective mass *M* subject to a static double well potential and driven by a monochromatic field of amplitude *A* and angular frequency Ω. The resulting time-dependent Hamiltonian for *S* reads

(25) $${\hat{H}}_{S}\left(t\right)=\frac{{\hat{p}}^{2}}{2M}+V\left(\hat{x}\right)-\hat{x}A\sin\left(\Omega t\right)={\hat{H}}_{0}-\hat{x}A\sin\left(\Omega t\right)\;,$$

where

(26) $$V\left(\hat{x}\right)=\frac{M^{2}\omega_{0}^{4}}{64\Delta U}{\hat{x}}^{4}-\frac{M\omega_{0}^{2}}{4}{\hat{x}}^{2}-\epsilon \hat{x}\;,$$

is the static potential parametrized by the quartic function of the particle’s coordinate $\hat{x}$.

**Figure 5:**
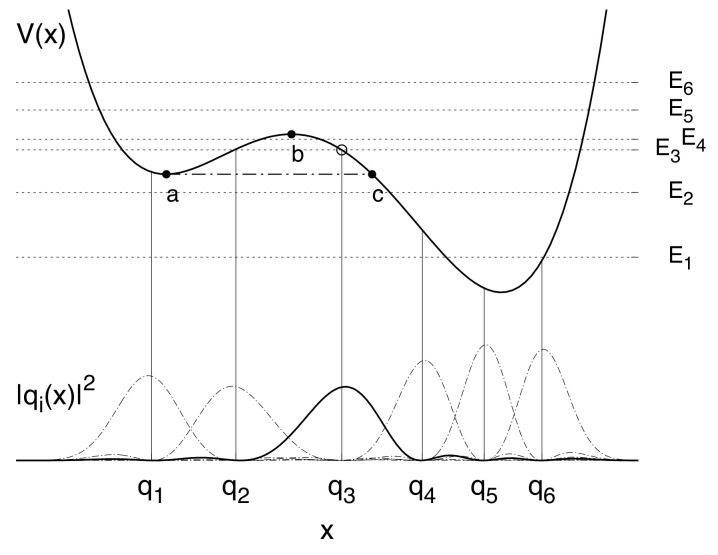
(Color online) Potential V(x) (Equation (26), with $\Delta U=1.4\;\hbar \omega_{0}$ and $\epsilon =0.27\sqrt{M\hbar \omega_{0}^{3}}$) and the first 6 energy levels (horizontal lines). In the lower part the probability densities $|q_{i}{\left(x\right)|}^{2}={\left|\langle x|q_{i}\rangle \right|}^{2}$ associated to the DVR eigenfunctions are shown, the initial state $|q_{3}\rangle$ being highlighted by a solid line. Vertical lines indicate the position eigenvalues in the DVR. The *metastable region* of the potential is to the left of the so-called *exit point c*.

The full time-dependent Hamiltonian is (see Equation (1))

(27) $$\hat{H}\left(t\right)={\hat{H}}_{S}\left(t\right)+{\hat{H}}_{B+SB}\;,$$

where ${\hat{H}}_{B+SB}$ is the bath Hamiltonian including the interaction with the system. In Equation (26), $\omega_{0}$ is the oscillation frequency around the potential minima, ϵ is a static bias and ΔU the barrier height at zero bias. Throughout the present work we scale all the physical quantities with $\omega_{0}$, which is of the same order of magnitude of the frequency spacing between ground state and the first excited energy level. We choose ϵ sufficiently large to get a configuration that, in the transient dynamics, is suitable for modeling the decay in a metastable potential, starting from a nonequilibrium condition. In the upper part of Figure 5, V(x) is shown for $\Delta U=1.4\;\hbar \omega_{0}$ and $\epsilon =0.27\sqrt{M\hbar \omega_{0}^{3}}$.

The bath spectral density function, which describes the frequency distribution of the reservoir’s oscillators and their coupling with the particle, is defined by [32,71,73,94]

(28) $$J\left(\omega \right)=\frac{\pi }{2}\sum_{j=1}^{N}\frac{c_{j}^{2}}{m_{j}\omega_{j}}\delta \left(\omega -\omega_{j}\right),$$

whose dimension is mass multiplied by frequency squared. In the general case of continuous bath the spectral density function is modeled as a power of ω, characterized by the exponent *s*, with an exponential cutoff at $\omega_{c}$

(29) $$J\left(\omega \right)=M\gamma_{s}{\left(\omega /\omega_{\mathrm{ph}}\right)}^{s-1}\omega e^{-\omega /\omega_{c}}.$$

The bath is said Ohmic for s=1. The so-called damping constant γ is a measure, in the continuous limit, of the system-bath coupling. The “phononic” reference frequency $\omega_{ph}$ [32] is introduced in such a way that γ has the dimension of a frequency also in the non-Ohmic case (s≠1). The exponential cut-off at high-frequency is introduced to avoid non-physical results as, for example, the divergence of the renormalization term in the Hamiltonian of Equation (1). The effect of the high frequency modes is taken into account by a redefinition of the particle’s bare mass, which is dressed by the high-frequency bath modes [32]. Here, we consider for the dissipative environment the Ohmic spectral density function J(ω) obtained from Equation (29) for s=1, $J\left(\omega \right)=M\gamma \omega e^{-\omega /\omega_{c}}$(see Equation (3)), while setting for the cutoff frequency $\omega_{c}=10\;\omega_{0}$. The coefficient γ, which has dimension of a frequency, provides a measure of the overall coupling between the system and the heat bath, whereas the couplings with the individual bath oscillators are given by the coefficients $c_{j}$ in Equation (1).

### 6.1. High-Frequency Driving

In the presence of a time-dependent driving the kernels do not depend anymore only on the difference $\tau =t-t^{\prime }$ and, consequently, after the integration over τ we have time dependent rates. However, if the frequency Ω of the monochromatic driving is sufficiently higher than any other frequencies (renormalized by the bath) of the system ($\Omega ≳\omega_{0}$), it is possible to take the average over one period T=2π/Ω [31,72], which gives for the rates of Equation (19)

(30) $$\Gamma_{jk}=\frac{\Omega }{2\pi }\int_{0}^{\frac{2\pi }{\Omega }}dt\int_{0}^{\infty }d\tau K_{jk}\left(t,t-\tau \right).$$

For j≠k, the kernels $K_{jk}$ read

(31) $$\begin{array}{cc} K_{jk}\left(t,t^{\prime }\right) & =2\Delta_{jk}^{2}e^{-q_{jk}^{2}Q^{\prime }\left(t-t^{\prime }\right)}\\ \times \cos\left[\zeta_{jk}\left(t,t^{\prime }\right)+q_{jk}^{2}Q^{\prime \prime }\left(t-t^{\prime }\right)\right], \end{array}$$

with the diagonal elements of the kernel matrix given by $K_{kk}\left(t,t^{\prime }\right)=-\sum_{n\neq k}K_{nk}\left(t,t^{\prime }\right)$, according to the conservation of probability. In Equation (31), $\Delta_{jk}\equiv \langle q_{j}|{\hat{H}}_{0}|q_{k}\rangle /\hbar$, $q_{jk}=q_{j}-q_{k}$, and the functions $\zeta_{jk}\left(t,t^{\prime }\right)$ are defined as the time integrals

(32) $$\begin{array}{ccc} \zeta_{jk}\left(t,t^{\prime }\right) & = & \int_{t^{\prime }}^{t}dt^{\prime \prime }\left[\left(\Delta_{jj}-\Delta_{kk}\right)-q_{jk}\left(A/\hbar \right)\sin\left(\Omega t^{\prime \prime }\right)\right]. \end{array}$$

Finally, in Equation (31) $Q^{\prime }$ and $Q^{\prime \prime }$ are respectively the real and imaginary part of the function Q(t), related to the bath correlation function L(t) (see Equations (7)–(9)). The master Equation (19), with rates given by Equation (30), describes the average effect of the high frequency driving on the time evolution of the populations $\rho_{jj}$ of the DVR states. The analytical solution of Equation (19) reads

(33) $$\rho_{jj}\left(t\right)=\sum_{n,k=1}^{M}S_{jn}{\left(S^{-1}\right)}_{nk}e^{\Lambda_{n}\left(t-t_{0}\right)}\rho_{kk}\left(t_{0}\right),$$

where S is the transformation matrix diagonalizing the rate matrix Γ, which has eigenvalues $\Lambda_{n}$. The smallest, in absolute value, of the nonzero eigenvalues determines the largest time-scale of the dynamics, the quantum relaxation time $\tau_{\mathrm{relax}}$ [72].

### 6.2. Escape Time for the Driven System

The transient dynamics of the driven system is analyzed by Equation (33), with the same nonequilibrium initial condition $\rho \left(0\right)=|q_{3}\rangle \langle q_{3}|$ used in the static case (23), that is with the particle initially prepared in the central region of the potential, on the right side of the barrier, between the maximum and the *exit point*, denoted by *c* in Figure 1. The escape time from the *metastable region* is defined, as in the static case (see Section 5), according to Reference [42]. Therefore, we calculate the population of the lower (right side) well, that is the cumulative population of the three DVR states from $|q_{4}\rangle$ to $|q_{6}\rangle$, $P_{\mathrm{right}}\left(t\right)=\sum_{j=4}^{6}\rho_{jj}\left(t\right)$. During the transient dynamics the cumulative population of the metastable well, coinciding with the overall population of $|q_{1}\rangle$ and $|q_{2}\rangle$, reaches a maximum and then, by tunneling through the potential barrier, decays settling down to a stationary value dependent on the temperature. We define the escape time τ from the metastable region of the potential, the region to the left of the exit point *c*, as the time the right well population takes to cross a threshold value *d*. The nonmonotonic behavior of τ as a function of γ and *T*, coupling coefficient and bath’s temperature, respectively, predicted in the static case (see Section 5), is robust against variations of the threshold around the value 0.9.

Here we set the threshold at d=0.95, which means that we consider the particle escaped from the metastable region when the probability to detect it in the lower (right) well is equal or greater than 95%. Note that, due to the incoherent relaxation described by Equation (33), once the threshold is crossed no oscillatory behavior of the populations occurs (no re-crossing of the threshold in the opposite direction). Therefore, if the particle crosses the threshold at time τ, the overall population of the metastable region is not going to be larger than 0.05 at later times.

We note that, in the static case, the metastable well can be thermally populated at the steady state. In this scenario no escape can occur if the threshold *d* is close to unity [85]. The same is true in the driven case for certain values of the frequency Ω, especially at large amplitudes *A*, whenever the left well population, namely the sum $P_{\mathrm{left}}=\rho_{11}+\rho_{22}$, is kept substantially above zero at the steady state by the presence of the driving.

### 6.3. Results and Discussion

In the absence of external driving, A=0, as γ increases, both the escape time τ and the relaxation time $\tau_{\mathrm{relax}}$ increase [85]. This holds until a critical value of $\gamma_{c}$, dependent on the temperature, is reached: by increasing further γ the escape time steeply diminishes whereas the relaxation time continues to increase monotonically.

In this section, we introduce $\bar{A}=A/\sqrt{M\hbar \omega_{0}^{3}}$ and $\bar{T}=k_{B}T/\hbar \omega_{0}$, the dimensionless driving amplitude and temperature, respectively.

**Figure 6:**
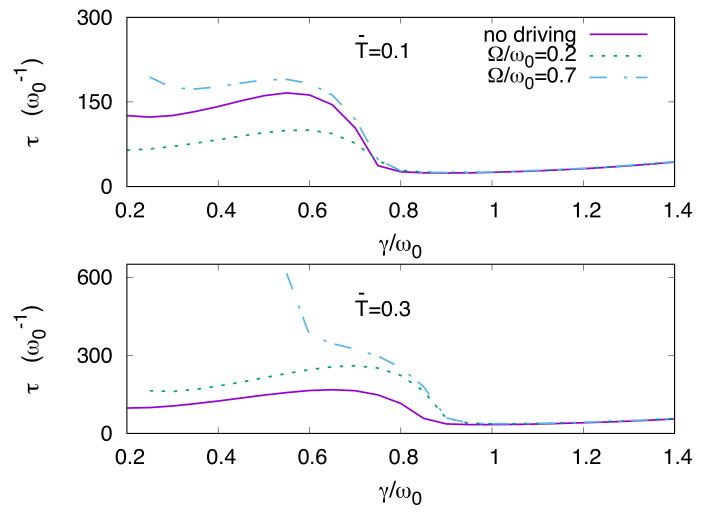
(Color online) Escape time vs. coupling strength for three driving settings, namely $\Omega /\omega_{0}=0,0.2,0.7$. Upper panel: Dimensionless temperature $\bar{T}=0.1$. At $\Omega /\omega_{0}=0.7$ no escape occurs for $\gamma /\omega_{0}≲0.25$. Lower panel: Dimensionless temperature $\bar{T}=0.3$. For $\Omega /\omega_{0}=0.2$ and $\Omega /\omega_{0}=0.7$, the escape occurs starting from $\gamma /\omega_{0}≲0.25$ and 0.55, respectively. For both panels the driving dimensionless amplitude is fixed at the value $\bar{A}=0.15$. Solid lines, in both panels, give the behavior in the absence of driving Ω=0. Here we introduced the dimensionless driving amplitude and temperature, $\bar{A}=A/\sqrt{M\hbar \omega_{0}^{3}}$ and $\bar{T}=k_{B}T/\hbar \omega_{0}$.

In Figure 6, the behavior of the escape time τ versus $\gamma /\omega_{0}$ for three values of frequency, namely $\Omega /\omega_{0}=0,0.2,0.7$, two different temperatures, that is $\bar{T}=0.1,0.3$, and dimensionless driving amplitude $\bar{A}=0.15$, is shown. At the lower temperature $\bar{T}=0.1$, all the curves show a nonmonotonic behavior, with a maximum, of τ as a function of the scaled coupling parameter $\gamma /\omega_{0}$. At the higher temperature $\bar{T}=0.3$, the same behavior occurs for the lower values of the scaled driving frequency $\Omega /\omega_{0}=0,0.2$, while a monotonic behavior is observed for the higher frequency value, i.e., $\Omega /\omega_{0}=0.7$. This monotonic behavior can be ascribed to the conjunct effect of thermal bath and driving force, which accelerates the escape process from the metastable region by increasing the coupling parameter γ. The maxima in the escape time imply that, at a given temperature, there is an optimal value of the coupling γ for which the depletion of the metastable region is delayed. According to the well known classical phenomenon [10,13,15,50,52,62], we address this feature as quantum noise enhanced stability (qNES) [85].

Moreover, a critical value of the coupling strength $\gamma_{c}$, dependent on the temperature, exists also in the presence of driving. The critical values of this coupling parameter in Figure 6 are found to be $\gamma_{c}/\omega_{0}≃0.75$ at $\bar{T}=0.1$ and $\gamma_{c}/\omega_{0}≃0.9$ at $\bar{T}=0.3$. We also observe that at the higher temperature, for $\Omega /\omega_{0}=0.7$, there is no escape up to $\gamma /\omega_{0}≃0.55$. At stronger dissipation the interaction with the heat bath forces the relaxation towards the lower well causing the depletion of the metastable well, which would be otherwise populated due to the combined effect of driving and thermal excitation. We note that $\gamma_{c}$ is larger at the higher temperature, indicating that at strong coupling, and in the presence of driving, the thermal excitations of the heat bath contrast the relaxation induced by the bath itself.

An interesting feature is the presence, at strong coupling and independently of the driving frequency, of a slow monotonic increase of the escape time τ for $\gamma /\omega_{0}>\gamma_{c}/\omega_{0}$, which is the signature of the quantum Zeno effect [86,87,88,89,90,91,92] (see also Section 5.1).

**Figure 7:**
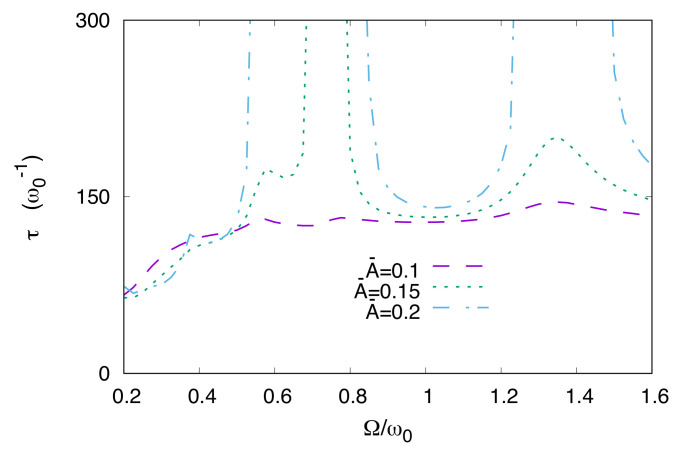
(Color online) Escape time vs. driving frequency for three values of the dimensionless driving amplitude $\bar{A}=A/\sqrt{M\hbar \omega_{0}^{3}}$, namely $\bar{A}=0.1,0.15,0.2$. Other parameters are $\gamma /\omega_{0}=0.2$ and $\bar{T}=0.1$. Here we introduced the dimensionless driving amplitude and temperature, $\bar{A}=A/\sqrt{M\hbar \omega_{0}^{3}}$ and $\bar{T}=k_{B}T/\hbar \omega_{0}$.

**Figure 8:**
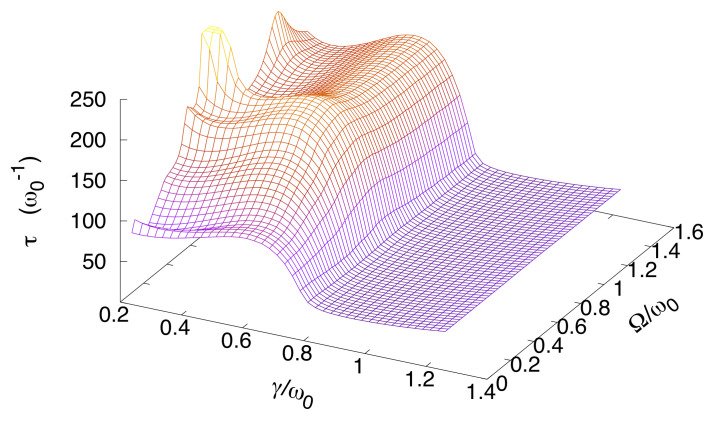
(Color online) Escape time as a function of the coupling strength and the driving frequency for dimensionless amplitudes $\bar{A}=0.15$. The dimensionless temperature is set to the value $\bar{T}=0.1$. Here we introduced the dimensionless driving amplitude and temperature, $\bar{A}=A/\sqrt{M\hbar \omega_{0}^{3}}$ and $\bar{T}=k_{B}T/\hbar \omega_{0}$.

Our main focus in this section is the investigation of the escape time as a function of the driving frequency and coupling strength. This is aimed at giving a systematic account of the delay and the quench of the escape shown in Figure 6, and at investigating how these effects are influenced by variations of the coupling with the environment. To this end, we fix the normalized damping coefficient at the lowest value used in our calculations, that is $\gamma /\omega_{0}=0.2$, and plot the escape time as a function of Ω for three different values of the dimensionless driving amplitudes $\bar{A}$, namely $\bar{A}=0.1,0.15,0.2$ (see Figure 7). Then we show the escape time as a function of Ω and γ, setting the dimensionless amplitude at the intermediate value, $\bar{A}=0.15$ (see Figure 8).

The plot τ vs. Ω, shown in Figure 7, is characterized by resonant peaks and dips whose magnitude is enhanced by increasing the driving amplitude, the escape being completely quenched for frequencies around $\Omega /\omega_{0}≃0.75$, when $A\geq 0.15\sqrt{M\hbar \omega_{0}^{3}}$. The effect is easily interpreted because the case $\Omega /\omega_{0}=0.75$ displays at the steady state a left-well population larger than (1−d), implying that $P_{\mathrm{right}}<d$. The frequencies for which τ is maximized (or no escape occurs, depending on the amplitude) roughly correspond to the energy separations $E_{3}-E_{2}\approx 0.6\hbar \omega_{0}$, $E_{4}-E_{2}\approx 0.8\hbar \omega_{0}$, and $E_{5}-E_{2}\approx 1.25\hbar \omega_{0}$, showing that a resonance phenomenon between the external driving and these frequencies occurs. For $\Omega \approx \omega_{0}$, a minimum of τ is visible, which is akin to the quantum resonant activation phenomenon [95] and noise suppression effect [24,25]. However, these noise-induced effects and the related relaxation dynamics have been observed by fixing the initial condition for the system in the metastable well. Here, the out-of-equilibrium initial condition makes the transient dynamics qualitatively different. Indeed, while $\tau_{\mathrm{relax}}$ is the time needed for the system to reach the equilibrium in the double well potential, the escape time (as defined in Section 5) is a relevant quantity for the transient dynamics, involving the crossing of the potential barrier and the emptying of the metastable well [85].

Peaks and dips in τ as a function of the driving frequency are smoothed out as γ increases, with τ becoming Ω independent as the coupling γ approaches the critical value $\gamma_{c}$.

This behavior is displayed in Figure 8, where the escape time is plotted as a function of $\Omega /\omega_{0}$ and $\gamma /\omega_{0}$ for $\bar{A}=0.15$. For $\gamma <\gamma_{c}$ the high frequency driving can delay or accelerate the escape, while for coupling strengths above the critical value $\gamma_{c}$ the escape time becomes frequency independent and quantum Zeno effect occurs [86,87,88,89,90,91,92].

The critical value $\gamma_{c}$ marks the transition to a dynamical regime, in which the tunneling mechanism of population transfer towards the *metastable region* is suppressed. This is because the tunneling dynamics becomes slow with respect to the depletion dynamics of the region where the particle is initially prepared. As a result, the probability of detecting the particle in the metastable well, starting from the initial condition (23), is always negligibly small as the population is directly transferred from $|q_{3}\rangle$ to the right well states. This effect is not captured by the relaxation time (see Section 6.1), which is independent of the initial condition and grows monotonically as γ increases.

## 7. Dissipative Dynamics in Sub-Ohmic and Super-Ohmic Regime

In this section, we study the bistable dynamics of a quantum particle coupled to an environment of which we vary the spectral density.

We assume the same algebraic spectral density function with exponential cutoff of Equation (29)

(34) $$J\left(\omega \right)=M\gamma_{s}{\left(\omega /\omega_{\mathrm{ph}}\right)}^{s-1}\omega e^{-\omega /\omega_{c}}.$$

**Figure 9:**
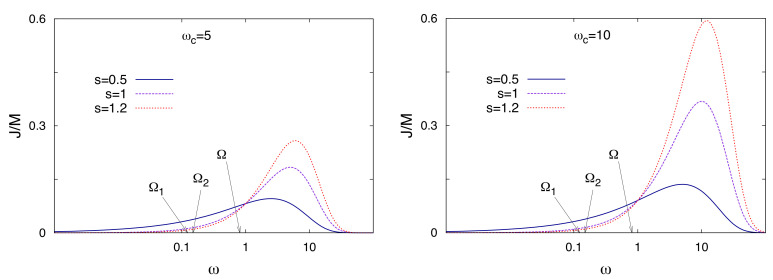
Bath spectral density function (see Equation (34)) as a function of ω in log scale. Three values of *s* are considered for each of the two values of the cutoff frequency $\omega_{c}=5$ (**left panel**) and $\omega_{c}=10$ (**right panel**). Frequencies are in units of $\omega_{0}$. The frequency spacings of the first and second energy doublet are $\Omega_{1}=0.123\;\omega_{0}$ and $\Omega_{2}=0.149\;\omega_{0}$, respectively. These two frequencies characterize the tunneling dynamics of the bistable system.

The bath is sub-Ohmic for 0<s<1, Ohmic for s=1 and super-Ohmic for s>1. The parameter $\gamma_{s}$, with dimension of frequency, is the system-bath coupling strength. Here we set the *phonon frequency* equal to the oscillation frequency around the potential minima of the potential profile, namely $\omega_{\mathrm{ph}}=\omega_{0}$. In Figure 9 we show the spectral density functions J(ω) in the sub-Ohmic, Ohmic, and super-Ohmic regimes for two values of the cutoff frequency. There, the density of low frequency modes is the highest in the sub-Ohmic regime. On the other hand, the density of high frequency modes, especially at large cutoff frequency, is the largest in the super-Ohmic regime.

We assume that the environment has a physical cutoff at $\omega_{c}$, which may be the Debye frequency of the medium in which the system is immersed. We consider two values of the cutoff frequency, namely $\omega_{c}=5\;\omega_{0},50\;\omega_{0}$. The bath modes with frequencies up to the frequency scale set by $\omega_{0}$ affect the particle dynamics through the quantum friction modeled by the Caldeira-Leggett Hamiltonian (Equation (1)) on the time scales of the intrawell motion or longer. The modes with higher frequencies affect the system dynamics by renormalizing the mass [71].

**Figure 10:**
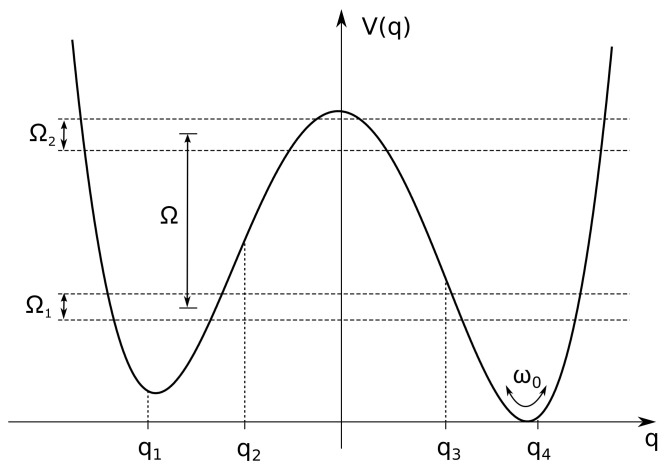
Potential $V_{0}$ with energy levels (horizontal lines) and DVR positions. The frequency $\omega_{0}$ is the oscillation frequency around the potential minima. The average inter-doublet frequency spacing, that is the characteristic frequency of the intrawell motion, is $\Omega =0.814\;\omega_{0}$. The frequency spacings of the first and second energy doublet are $\Omega_{1}=0.123\;\omega_{0}$ and $\Omega_{2}=0.149\;\omega_{0}$, respectively. These two frequencies characterize the tunneling dynamics of the bistable system.

Moreover, we consider the dynamics of the dissipative bistable system, beyond the TLS approximation, in a temperature regime in which the presence of the second energy doublet cannot be neglected. We therefore describe the system dynamics by taking into account the first four energy levels (see Figure 10). We investigate the quantum dissipative dynamics by using a nonperturbative generalized master equation with approximated kernels, derived within the Feynman-Vernon influence functional approach [68,74,96]. We study the reduced dynamics by varying the exponent *s* in the crossover from the sub-Ohmic (s<1) to the super-Ohmic (s>1) dissipation regime. We consider also the effects of changing the cutoff frequency $\omega_{c}$, i.e., the contribution of the high-frequency modes to the open dynamics. Recently, for a quantum dot modeled as a TLS interacting with bosonic and electronic environments at zero temperature, a similar study on the effects of varying the spectral density function from sub-Ohmic to super-Ohmic regime has been performed [97].

### Dynamics and Relaxation Times

In this section, we show the results for the dynamics obtained by numerical integration of the GME (Equation (16)) with the NIBA kernels given by Equation (31) and initial time set to $t_{0}=0$.

Calculations are performed by varying *s*, the exponent of ω in the spectral density function J(ω), in the range 0.5≤s≤1.2, and for different cutoff frequencies $\omega_{c}$ (see Equation (34)). We consider two temperature values T=0.2,0.5 in unit of $\hbar \omega_{0}/k_{B}$ and fix the coupling strength to the value $\gamma_{s}\equiv \gamma =0.1\;\omega_{0}$. Throughout this section the system is assumed to be initially in the localized state $|q_{1}\rangle$ belonging to the left well (see Figure 10).

**Figure 11:**
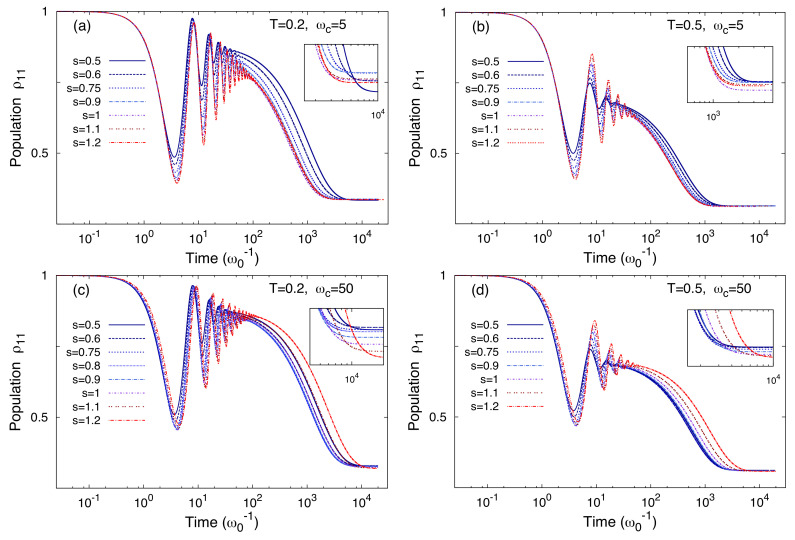
Time evolution of the population $\rho_{11}$ of the state $|q_{1}\rangle$ for different values of *s*, at two cutoff frequencies $\omega_{c}=5$ (panels (**a**) and (**b**)) and $\omega_{c}=50$ (panels (**c**) and (**d**)) and for two temperatures T=0.2 (panels (**a**) and (**c**)) and T=0.5 (panels (**b**) and (**d**)). Insets: Relaxation towards the stationary values of $\rho_{11}$. The coupling strength $\gamma_{s}$ is fixed to the value $0.1\;\omega_{0}$. Temperatures and frequencies are in unit of $\hbar \omega_{0}/k_{B}$ and $\omega_{0}$, respectively.

In Figure 11 the time evolution of the population $\rho_{11}$ of the state $|q_{1}\rangle$ is shown at two temperatures and for two values of the cutoff frequency $\omega_{c}$. The time evolutions of $\rho_{11}$ display damped intrawell oscillations ending up in a metastable intrawell equilibrium state which relaxes further towards a stationary configuration over a much larger time scale. The presence of these two different time scales reflects the two different frequency scales of tunneling and intrawell motion in the bare system. Moreover the tunneling dynamics is strongly damped due to the distance between states in different wells. This is because the prefactor ${\left(q_{i}-q_{j}\right)}^{2}$ multiplying $\gamma_{s}$ in the exponent of the NIBA kernels (Equation (31)) yields a large effective coupling. We also observe that, in each panel of Figure 11, the intrawell oscillations are slower for higher *s*. The effect is more evident at the higher value of the cutoff frequency. This can be ascribed to a larger renormalized mass due to the stronger presence of high frequency modes, especially for the higher cutoff frequency [71].

Further, for both cutoff frequencies, the higher is *s* the less the oscillations are damped. This is because, on the time scale of the intrawell motion, the bath modes contributing to the quantum friction are those with ω≲Ω, which are denser at lower *s*. Note also that, by varying *s*, the long time dynamics of $\rho_{11}$ has different behaviors for the two cutoff frequencies. In particular, for $\omega_{c}=5\;\omega_{0}$ (upper panels of Figure 11) the relaxation is faster at high *s*, while for $\omega_{c}=50\;\omega_{0}$ (lower panels) is faster at low *s*. This is also shown in the insets of Figure 11 along with the asymptotic behavior.

**Figure 12:**
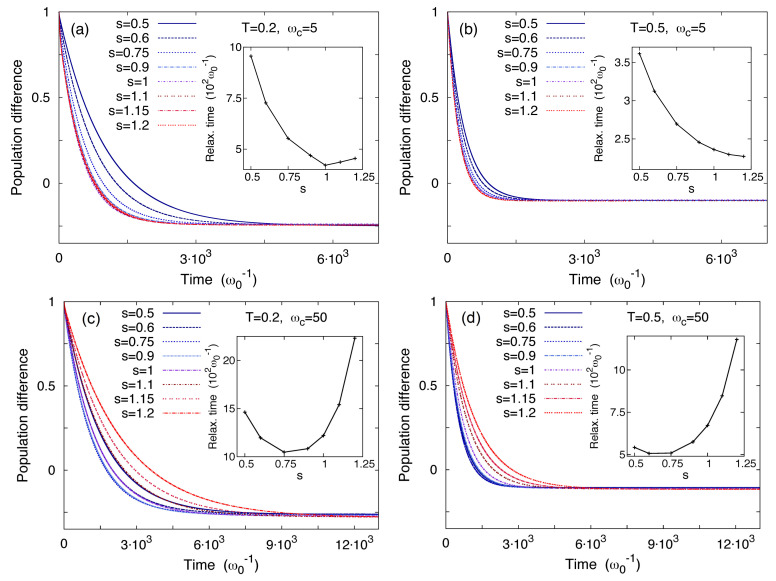
Time evolution of the population difference $P_{L}-P_{R}$ for different spectral densities (0.5≤s≤1.2) at temperatures T=0.2 (panels (**a**) and (**c**)) and T=0.5 (panels (**b**) and (**d**)) and at cutoff frequencies $\omega_{c}=5$ (panels (**a**) and (**b**)) and $\omega_{c}=50$ (panels (**c**) and (**d**)). Insets: Relaxation times $\Lambda_{min}^{-1}$ as a function of *s*. The coupling strength is $\gamma_{s}=0.1\;\omega_{0}$. Temperatures and frequencies are in units of $\hbar \omega_{0}/k_{B}$ and $\omega_{0}$, respectively.

The features of the relaxation towards the stationary configuration are displayed in Figure 12, where the time evolution of the population difference $P_{L}-P_{R}$, where $P_{L(R)}=\rho_{11(33)}+\rho_{22(44)}$, is shown for two values of the temperature and cutoff frequency, along with the relaxation time $\Lambda_{\min}^{-1}$ as a function of *s* . The $\omega_{c}$-dependent minima in the relaxation time as a function of the exponent *s*, shown in the insets of Figure 12, emerge as the result of two competing mechanisms. On the one hand, by lowering the exponent *s* the density of low-frequency modes ($\omega ∼\Omega_{1,2}$) of the bath is increased (see Figure 10). These modes act on the time scales of the tunneling $\Omega_{1,2}^{-1}$ contributing to the friction exerted by the heat bath. As a consequence, by moving towards smaller values of *s*, the dissipation is enhanced and the tunneling is hampered. On the other hand, the mass renormalization term increases with *s* slowing down the relaxation due to the increased inertia of the system [71]. These two competing behaviors yield the minima in the relaxation times. This physical picture is confirmed by the fact that for large $\omega_{c}$, where the mass renormalization effect is stronger, the minimum moves towards lower values of *s*.

By a suitable combination of DVR states it is possible to construct initial states of our four-level system corresponding to the left and right state (|L〉, |R〉) involving only the lower energy doublet. If the temperature is low enough, the time evolution of a system prepared in one of these sates does not display intrawell oscillations, which involve transitions to higher energy states induced by the heat bath, and a treatment based on the two-level system approximation is appropriate [73]. However, at the temperatures considered in our work, especially at $T=0.5\;\omega_{0}$, where $\omega_{0}$ sets the order of magnitude of the separation between the first and second energy doublet, intrawell oscillations are expected to be activated regardless of the initial condition. These intrawell oscillations undergo the damping effects described in our analysis. Of course, the transient dynamics for our initial condition is expected to differ from that resulting from a preparation which entails the presence of coherences, with the inhomogeneity term in Equation (13) playing a role in the transient. Nevertheless, due to the exponential suppression of this contribution, the relaxation times and the stationary values of the populations are not affected by the initial condition. Therefore, apart from the details of the transient dynamics, the results of our analysis are not modified by initial preparations which involve the presence of the inhomogeneity term.

## 8. Conclusions

In this paper, we reviewed some results on the escape processes from quantum metastable states strongly interacting with an Ohmic thermal bath. The theoretical analysis is performed by a non-perturbative technique based on the real-time path integral approach of the Feynman-Vernon influence functional. In particular, in the absence of driving, we found that the escape time exhibits a nonmonotonic behavior, with a maximum, for increasing values of the damping. This indicates the presence of a noise induced phenomenon, which we name quantum noise enhanced stability (qNES). Moreover, the behaviour of the escape time shows a nonmonotonic behaviour, characterized by a minimum at the resonance with the tunneling splitting, as a function of the temperature [85].

One novelty of this study is that we analyze the out-of-equilibrium dynamics of a quantum particle, by fixing a threshold whose crossing indicates that the particle escaped from the metastable region, and by studying an escape time, suitably defined, as a function of the coupling strength (damping) and the temperature of the heat bath.

In the presence of a periodical external driving, within the same framework of the dissipative quantum dynamics of the asymmetric bistable system, we analyzed the escape time from the metastable region as a function of the driving frequency and the parameters of the thermal bath. We found a nonmonotonic behavior of the escape time from the metastable region as a function of the temperature, frequency of the driving, and thermal-bath coupling, which indicates the presence of a qNES phenomenon in the system investigated. Moreover, we observed the presence of resonant peaks and dips for lower values of the coupling coefficient γ. For increasing values of γ, after the maximum, a steep decrease of the escape time is observed and the escape time becomes frequency-independent. More in detail, a value $\gamma_{c}$ exists such that for $\gamma >\gamma_{c}$ the behavior of the escape time highlights the presence of a transition, occurring at this critical value $\gamma_{c}$, between two qualitatively different dynamical regimes of the metastable system.

Finally, the role of different spectral densities, either in sub-Ohmic or super-Ohmic dissipation regime and different cutoff frequencies, on the the relaxation dynamics from the quantum metastable state was investigated. We found that for higher values of the exponent *s* the populations undergoes less damped intrawell oscillations and the relaxation time exhibits a minimum at a value of *s* depending on the cutoff frequency [71]. The results obtained indicate that, in the crossover dynamical regime characterized by damped *intrawell* oscillations and incoherent tunneling, the spectral properties of the thermal bath influence non-trivially the time scales of the relaxation dynamics from the metastable state.

The phenomenon of quantum noise enhanced stability (qNES) can be observed within existing experimental frames such as superconducting qubits [75] and optical trapping [98,99]. Moreover, our results on the escape dynamics from metastable states can be useful to control the stability of trapped particle in optical communication, and to engineer dissipative environments for superconducting and quantum computation devices [100,101,102].
